# Characterization of Flower-Bud Transcriptome and Development of Genic SSR Markers in Asian Lotus (*Nelumbo nucifera* Gaertn.)

**DOI:** 10.1371/journal.pone.0112223

**Published:** 2014-11-07

**Authors:** Weiwei Zhang, Daike Tian, Xiu Huang, Yuxian Xu, Haibo Mo, Yanbo Liu, Jing Meng, Dasheng Zhang

**Affiliations:** 1 Shanghai Chenshan Plant Science Research Center, Chinese Academy of Sciences, Chenshan Botanical Garden, Shanghai, China; 2 College of life and Environmental Sciences, Shanghai Normal University, Shanghai, China; 3 College of Horticulture, Northeast Agricultural University, Harbin, China; Wuhan Botanical Garden, Chinese Academy of Sciences, Wuhan, China

## Abstract

**Background:**

Asian lotus (*Nelumbo nucifera* Gaertn.) is the national flower of India, Vietnam, and one of the top ten traditional Chinese flowers. Although lotus is highly valued for its ornamental, economic and cultural uses, genomic information, particularly the expressed sequence based (genic) markers is limited. High-throughput transcriptome sequencing provides large amounts of transcriptome data for promoting gene discovery and development of molecular markers.

**Results:**

In this study, 68,593 unigenes were assembled from 1.34 million 454 GS-FLX sequence reads of a mixed flower-bud cDNA pool derived from three accessions of *N. nucifera*. A total of 5,226 SSR loci were identified, and 3,059 primer pairs were designed for marker development. Di-nucleotide repeat motifs were the most abundant type identified with a frequency of 65.2%, followed by tri- (31.7%), tetra- (2.1%), penta- (0.5%) and hexa-nucleotide repeats (0.5%). A total of 575 primer pairs were synthesized, of which 514 (89.4%) yielded PCR amplification products. In eight *Nelumbo* accessions, 109 markers were polymorphic. They were used to genotype a sample of 44 accessions representing diverse wild and cultivated genotypes of *Nelumbo*. The number of alleles per locus varied from 2 to 9 alleles and the polymorphism information content values ranged from 0.6 to 0.9. We performed genetic diversity analysis using 109 polymorphic markers. A UPGMA dendrogram was constructed based on Jaccard’s similarity coefficients revealing distinct clusters among the 44 accessions.

**Conclusions:**

Deep transcriptome sequencing of lotus flower buds developed 3,059 genic SSRs, making a significant addition to the existing SSR markers in lotus. Among them, 109 polymorphic markers were successfully validated in 44 accessions of *Nelumbo*. This comprehensive set of genic SSR markers developed in our study will facilitate analyses of genetic diversity, construction of linkage maps, gene mapping, and marker-assisted selection breeding for lotus.

## Introduction

Asian lotus *(Nelumbo nucifera* Gaertn.), also called sacred lotus, is a diploid eudicot, that lies at the base of the angiosperm linage [1], and has an estimated genome size of 929 Mb [2]. Lotus is a perennial aquatic herbaceous plant that has been extensively cultivated as an ornamental plant for its magnificent flowers, as a food crop for its nutritive rhizomes and seeds, and as a source of herbal medicines. Other than its agricultural and medicinal importance, sacred lotus has many unique biological features. The most notable examples are seed longevity and ‘lotus effect’ or the unusal aquaphobic nature of the leaves. Lotus has also evolved as a unique cultural and religious icon in both Buddhism and Hinduism [3].

Lotus belongs to the family Nelumbonaceae, which consists of one genus *Nelumbo* Adans. with only two species, *N. nucifera* Gaertn. (Asia, north Australia and south Russia) and *N. lutea* Willd. (North America and Northern South America) [3]–[6]. The two species differ in external morphologies (plant size, leaf size, flower color and form, etc.) [6], [7] and have significant genetic differences [7]–[15], but there is no interspecific hybridization barrier and the offspring are viable and fertile [4]. Rich germplasm resources have been developed from natural and artificial hybrids within or between the two species. More than 800 lotus cultivars have been recorded in China [16], and are classified into three categories according to the morphological characteristics and agricultural utilization: flower, rhizome and seed [6], [15]. With many attractive floral characteristics (e.g., petal color, petal number, flower size, flower color, flower form, flowering period, and fragrance, etc.), the flower lotus has been studied and discussed more extensively than the rhizome or seed lotus. These floral characteristics are often used as the standards for classification, and always attract the attention of lotus breeders for germplasm improvement associated with ornamental and economic values. Efforts by traditional breeding methods have produced many lotus cultivars with diverse flower colors (red, pink, white, light yellow, multicolor), different flower forms (single, semidouble, double, duplicate, thousand-petalled), and an extended flowering period [6], [16]. However, the molecular mechanisms underlying formation of these attractive floral features remain unknown. Therefore, understanding the processes that regulate the formation and development of flower characteristics is of particular importance, especially at the molecular level. Such knowledge will facilitate the improvement of ornamental characteristics and the directional molecular breeding for lotus in the future.

Currently, several types of lotus genomic resources are available, including a draft genome sequence [3], expressed sequence tags (ESTs) [3], [17]–[18], and one linkage map [7]. The completion of the lotus genome will permit evolutionary and comparative genomics, and identification of key genes of biological and economic interests. Complementary to the whole genome sequence, ESTs present an alternative valuable resource for research because these provide the comprehensive information regarding the transcriptome for specific biological processes [19]. Large numbers of ESTs with broad coverages are invaluable for accelerating gene discovery and identification [19]–[22], comparative genomics [23], [24], large-scale expression analysis [25], development of molecular markers [26]–[29], and phylogenetic studies [30], [31]. Recently, an increasing number of EST datasets have become available for multiple organisms, but relatively few ESTs are available for lotus. Transcriptome sequence data for seven lotus tissues including root, leaf, petiole, embryonic axis, rhizome internode, rhizome apical meristem and rhizome elongation zone have been deposited in the National Center for Biotechnology information (NCBI) database (http://www.ncbi.nlm.nih.gov/sra/?term=nelumbo). However, transcriptome sequences of flower-bud tissues are not publicly available.

Simple sequence repeat (SSR) markers are very useful for a wide range of applications in plant genetics and breeding because of their abundance, random distribution within genomes, co-dominant multi-allelic nature, high reproducibility and polymorphism [32], [33]. There are two classes of SSRs, genomic SSRs (located in non-coding genomic regions) and genic SSRs (found in expressed sequences). Genic SSRs generally are more evolutionarily conserved within and across related species [34]. Additionally, genic SSRs may represent the specific transcriptional regions that contribute to important agronomic traits [34], [35]. Therefore, genic SSR are useful tools to facilitate gene cloning, map construction, and marker-assisted selection (MAS) breeding. So far, a limited number of SSRs, including genomic SSRs (less than 500) from the previous studies [7], [9], [11], [13]–[15], [36]–[38], and genic SSRs (only 39) from ESTs [7], [11], have been developed for lotus. Therefore, there is a need and opportunity for developing additional SSR markers to be used for lotus molecular breeding.

The following is a description of the generation, assembly and annotation of a transcriptome-derived expressed sequence dataset based on the 454 GS-FLX Titanium sequencing data from the young flower-buds of three accessions of *N. nucifera*. To the best of our knowledge, this is the first report of the transcriptome of the lotus flower -bud, and it will facilitate gene cloning and functional studies of genes involved in lotus growth and flower development. Additionally, we developed a comprehensive set of genic SSR markers and illustrated their utility within 44 accessions of *Nelumbo*. These genic SSR markers will greatly enrich the number of SSRs markers and will facilitate gene mapping, linkage map construction, genetic diversity analysis and MAS breeding in lotus.

## Results

### Transcriptome sequencing and assembly

**Figure 1:**
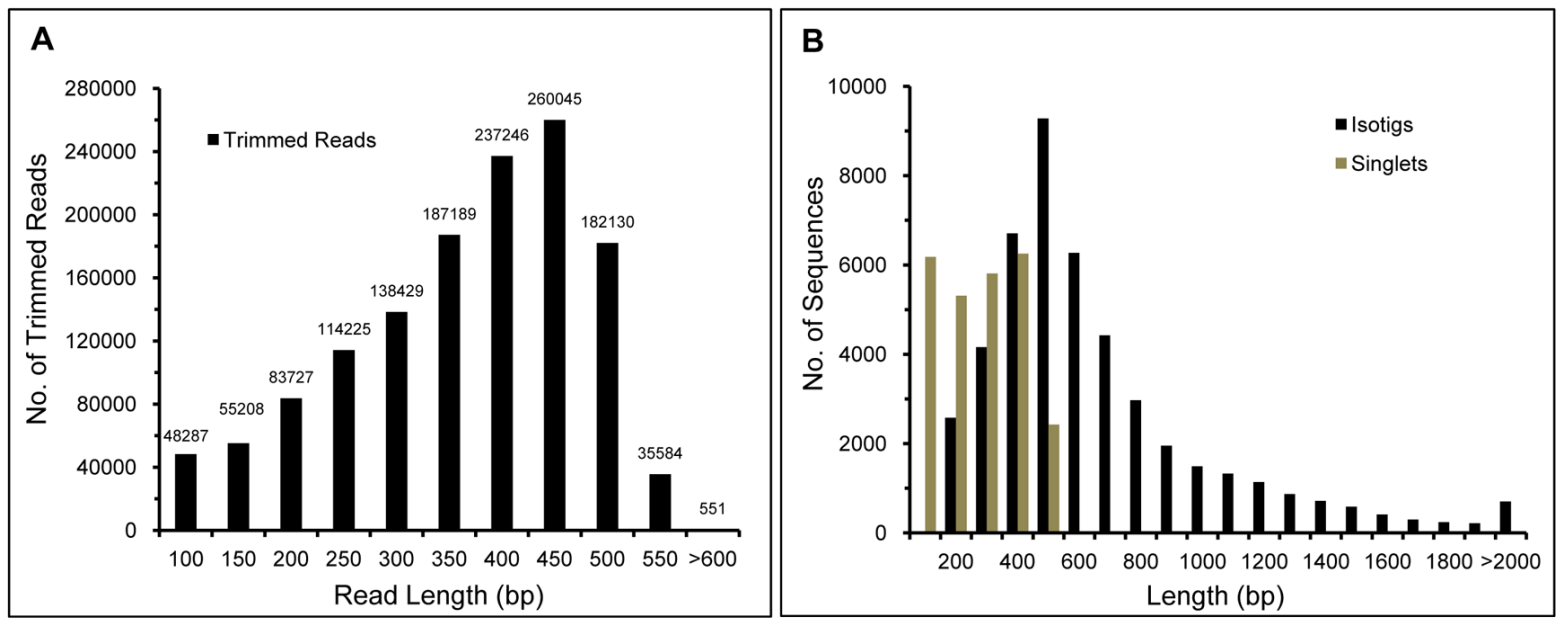
Size distribution of reads, isotigs and singlets by the transcriptome sequencing. (A) Length distribution of the sequencing reads after trimming low-quality reads. (B) Size distribution of the isotigs and singlets. The longest isotig was 7,170 base pairs.

A total number of 1,407,753 raw reads with an average length of 370 bp were generated by high-throughput sequencing of a mixed flower bud cDNA pool from three accessions of *N. nucifera* (**Table 1**). After removing low-quality reads including adapters, primers sequences, and short sequences (<50 bp) by a stringent trimming process, 1,342,621 clean reads (87.2%) were obtained with an average length of 338 bp (**Table 1**, **Figure 1a**). The total length of clean reads was about 454 million bases (453,913,177). Using CAP3 and Newbler software, the clean and qualified reads were assembled *de novo* into 46,348 isotigs with 25,998 remaining as singletons, for a total of 72,346 unique sequences. More than half of the total assembled length of isotigs was> 700 bp (N50  =  703 bp) (**Table 1**). The size distribution of isotigs and singletons is shown in **Figure 1b**.

**Table 1:** Raw reads and assembled data information by transcriptome sequencing.

Raw reads		Trimmed reads		Assembly data	
Total number of Reads	1,407,753	Total clean reads	1,342,621	Total number of isotigs	46,348
Total length of Reads (bp)	520,201,137	Total length (bp)	453,913,177	Total length of isotigs (bp)	28,734,639
Minimum Read length (bp)	19	Minimum Read length (bp)	50	Isotig N50 (bp)	703
Maximum Read length (bp)	1013	Maximum Read length (bp)	608	Number of singletons	25,998
Mean Read length (bp)	370	Mean Read length (bp)	338	Total number of unigenes	68,593
GC content (%)	44.99	GC content (%)	44.91	Mean unigene length (bp)	506

**Figure 2:**
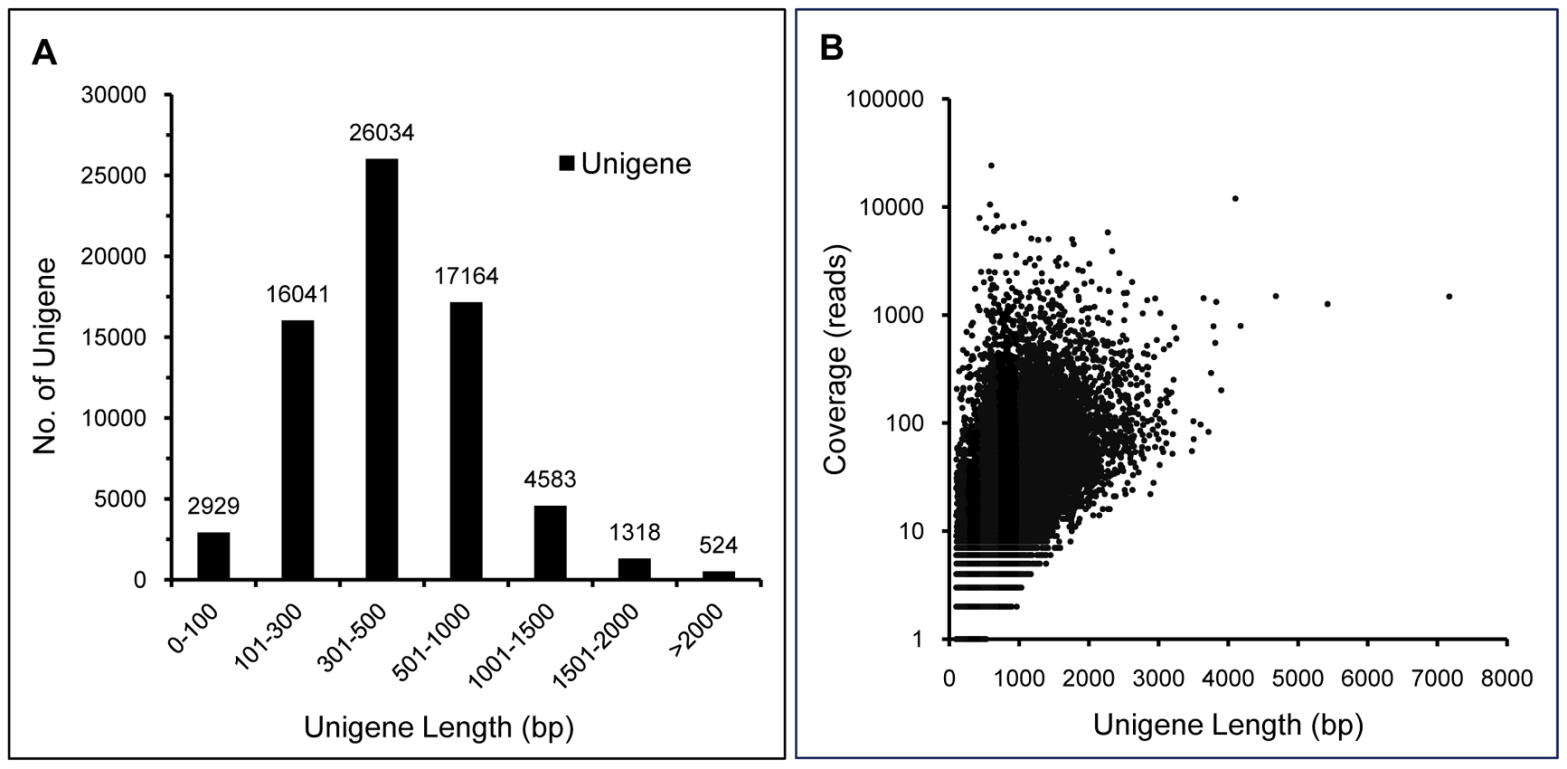
Distribution of the unigene length and coverage depth by the transcriptome assembly. (A) Length distribution of the assembled unigenes. (B) A density scatter-plot showing the relationship between unigene length and coverage. X-axis and y-axis labels refer to the unigene lengths and the read-depth coverage for assembled unigenes, respectively.

A total number of 68,593 unigenes with an average length of 506 bp were obtained in the study by combining and clustering the assembled unique sequences with CD-HIT 4.0 (**Table 1**). The length of 45,004 (65.6%) unigenes ranged from 100 to 500 bp, 17164 (25.0%) from 500 to 1000 bp, and 6,425 (9.4%) were more than 1000 bp in length (**Figure 2a**). The length of a unigene was related to the number of assembled sequences. The unigene length exhibited a gradual increase with the increasing read-depth (**Figure 2b**).

### Functional annotation of the transcriptome

**Figure 3:**
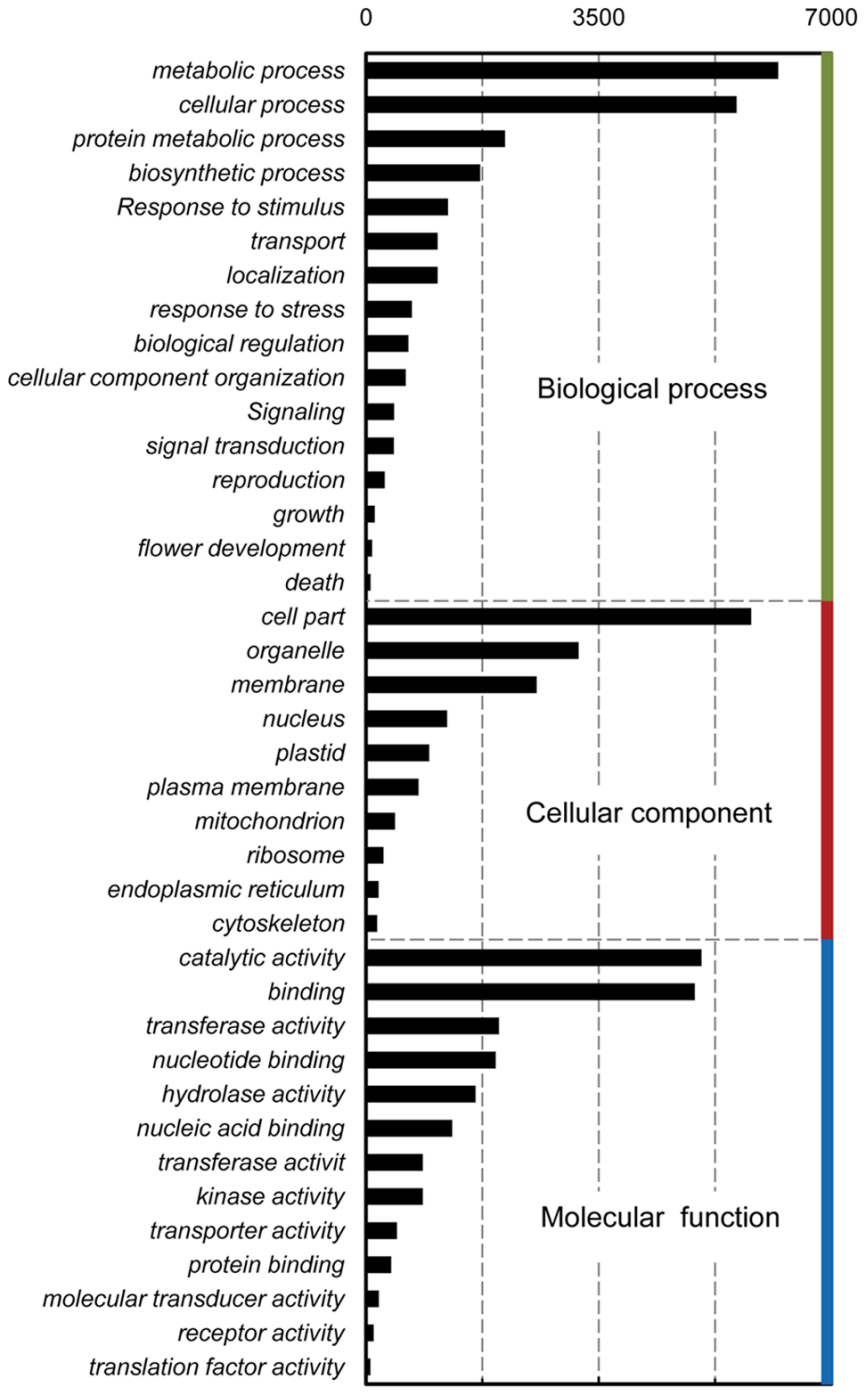
Functions classification of the annotated unigenes. Results are grouped by three main functional categories, Biological process, Cellular component and Molecular function. The top abscissa indicates the number of unigenes in a category. Bars show the number of assignments of protein matches to each GO term using BLASTx.

BLASTx was used to annotate the putative unigenes based on a sequence similarity search against the NCBI Non-Redundant protein database. Among the 68,593 unigenes, 34,341 (50.1%) unigenes, including 27,786 isotigs and 6,655 singletons, aligned with proteins of other species. Over 39% (27,193) had high similarities (e value ? 1e^−5^ and percentage of identical match ? 50%) to known sequences. However, homologous sequences could not be identified for about one half of the unigenes, indicating that these potential novel transcripts may play specific roles in the floral development of *N. nucifera*. Gene ontology assignments were applied and the functions of the unigenes were classified into a diverse range of functional classes (**Figure 3**).

**Figure 4:**
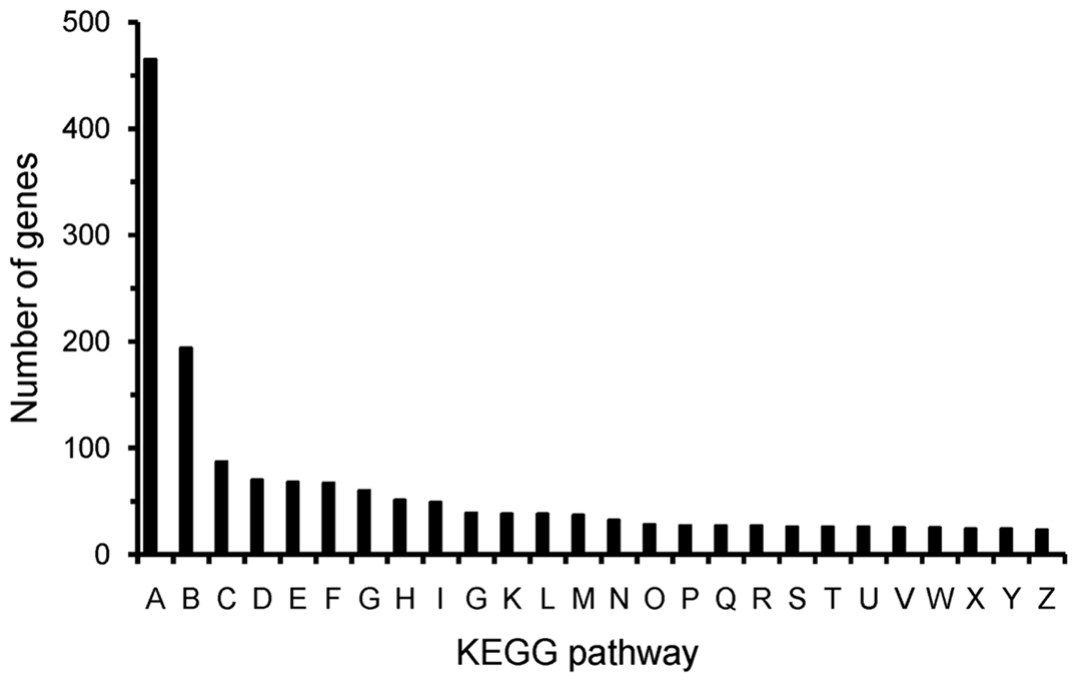
Histogram presentation of KEGG classification. A–Z are the top 26 KEGG pathways. The y-axis indicates the number of all genes with pathway annotation. The x-axis indicates the KEGG pathway. A, Metabolic pathways; B, Biosynthesis of secondary metabolites; C, Microbial metabolism in diverse environments; D, RNA transport; E, Ribosome; F, Spliceosome; G, Protein processing in endoplasmic reticulum; H, Purine metabolism; I, Pyrimidine metabolism; J, Oxidative phosphorylation; K, Ubiquitin mediated proteolysis; L, Cell cycle; M, Cell cycle-yeast; N, RNA degradation; O, Lysosome; P, Meiosis-yeast; Q, Endocytosis; R, Nucleotide excision repair; S, mRNA surveillance pathway; T, Proteasome; U, DNA replication; V, N-Glycan biosynthesis; W, Aminoacyl-tRNA biosynthesis; X, Glycolysis/Gluconeogenesis; Y, Amino sugar and nucleotide sugar metabolism; Z, Peroxisome.

Pathway-based analysis for the transcriptome of lotus flower bud is helpful to further understand the biological functions and genes interactions. A total of 13,536 genes were assigned to 232 different pathways in the KEGG database (Kyoto Encyclopedia of Genes and Genomes), and the top 26 KEGG pathways are shown in **Figure 4**. The pathways with most representation were ‘Metabolic’ and ‘Biosynthesis of secondary metabolites’ (**Figure 4**), which indicates that the diverse metabolic processes are active and a variety of metabolites are synthesized in the flower bud of *N. nucifera*.

### Transcripts related to flower development

A total of 152 putative homologs related to flower development genes were identified, and they were involved in eight pathways such as the anthocyanin biosynthesis (65), carotenoid biosynthesis (15), specification of floral organ identity (12), photoperiod (21), vernalization (5), gibberellic acid (3), ethylene biosynthesis (17), and other genes of flower development (14) (**Table S1**). Identification of these genes will aid the understanding of the molecular mechanisms involved in the formation and development of important flower characteristics of lotus in the future, especially in the colorants form of flower or fruit, flowering-time, floral organ identity, flower forms, and flower senescence etc. EST sequences of all 152 genes identified in the study are listed in **Dataset S1**.

### Identification of EST-SSR markers

**Figure 5:**
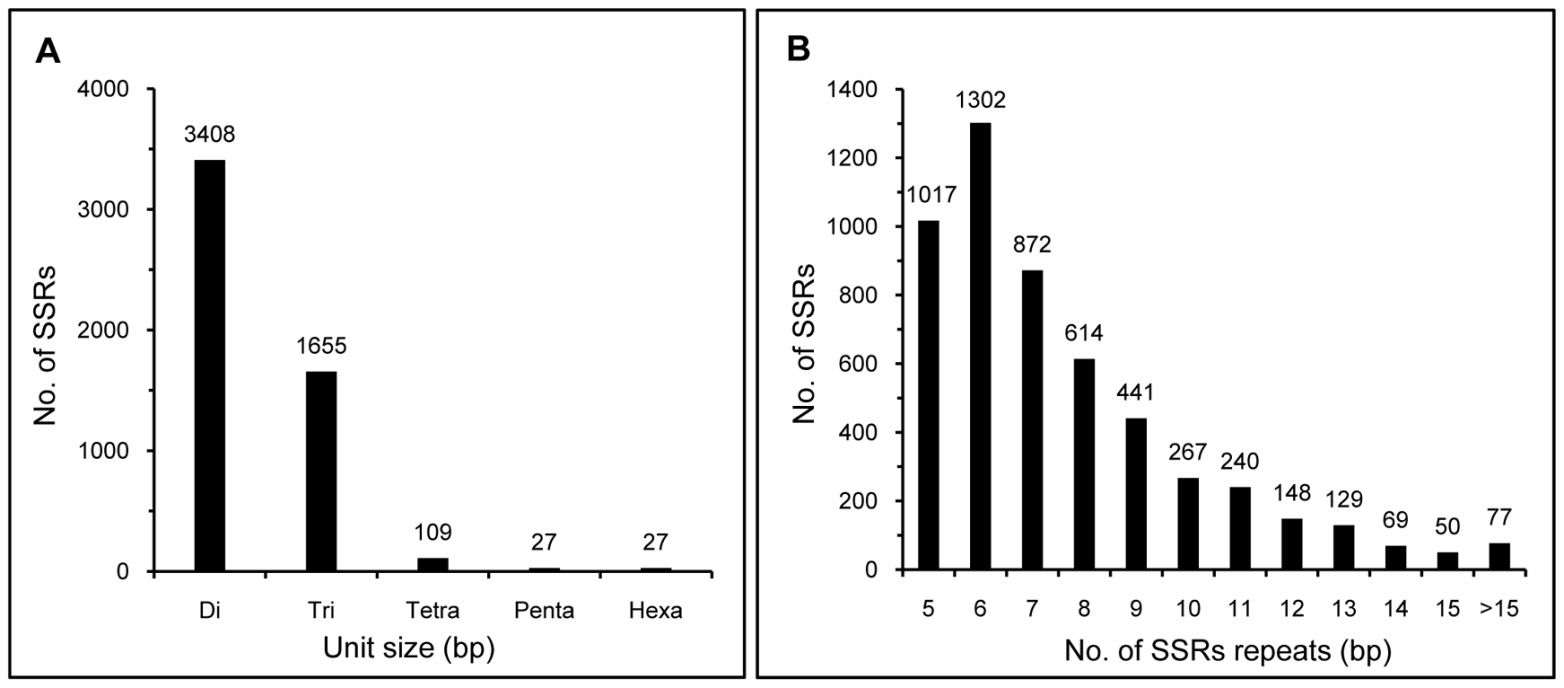
Frequency distribution of the SSRs identified in transcriptome dataset. (A) Distribution of the total number of EST-SSRs in different classes of repeat type. Di-, tri-, tetra-, penta- and hexa-nucleotide repeats were analyzed. (B) Distribution of the number of SSRs repeats. The number of repeats ranged from 5 to 39.

Using a perl script known as MISA, we identified 6,086 SSR loci from 68,593 unigenes generated in this study, with an average of one SSR locus per 5.7 kb DNA. Of these, 550 unigenes (10.5%) contained more than one SSR and 339 (6.5%) contained compound SSRs with more than one repeat type (**Table 2**). SSRs with mononucleotide repeats were not considered in this study, and the remaining 5,226 SSRs included di-, tri-, tetra-, penta-, and hexa-repeats. Di-nucleotide repeat motifs were the most abundant type, with a frequency of 65.2% (3,408), followed by tri- (31.7%, 1,655), tetra- (2.1%, 109), penta- (0.5%, 27) and hexa-nucleotide repeats (0.5%, 27) (**Figure 5a**). Frequencies of SSRs with different numbers of tandem repeats are shown in **Figure 5b**. The number of SSR repeats ranged from 5 to 39, and SSRs with six tandem repeats (24.9%) were the most abundant, followed by five tandem repeats (19.5%), seven tandem repeats (16.7) and eight random repeats (11.8%), respectively. Motifs that showed more than 15 repeats were rare, with a frequency of less than 1.5%. The top 10 abundant SSR repeat motifs with different levels of repeats are shown in **Table 3**. C/G-rich (0.5%) motifs were rare in our database.

**Table 2:** Characteristics of SSRs identified in transcriptome dataset.

Total number of sequences examined	68,593
Total size of examined sequences (kbp)	34,682
Total number of identified SSRs	6,086
Number of SSR containing sequences:	5,482
Number of sequences containing more than 1 SSR	550
Number of SSRs present in compound formation	339
Frequency of SSR in transcriptome	1/5.7 kbp

**Table 3:** Distribution of the top ten abundant SSR motifs with different levels of repeats in transcriptome.

No.	Repeats motif	Number of repeats units													
		5	6	7	8	9	10	11	12	13	14	15	>15	Total	%
1	GA/TC	-	389	321	223	187	106	106	59	69	24	19	33	1536	29.39
2	AG/CT	-	343	299	247	176	121	100	76	47	35	21	26	1491	28.53
3	GAA/TTC	128	67	30	9	12	8	6	3	5	1	2	5	276	5.28
4	AGA/TCT	111	54	17	12	11	7	7	4	1	3	2	6	235	4.50
5	AAG/CTT	92	47	17	11	4	4	2	-	1	1	1	3	183	3.50
6	AT/TA	-	64	38	18	12	3	2	-	-	-	-	-	137	2.62
7	AC/GT	-	42	31	21	14	2	4	2	3	-	2	1	122	2.33
8	CA/TG	-	45	35	19	7	3	3	1	2	2	1	1	119	2.28
9	ATC/GAT	58	34	4	9	-	1	1	1	-	1	-	-	109	2.09
10	CAG/CTG	43	21	1	2	2	3	-	-	-	-	-	-	72	1.38

### Development and evaluation of EST-SSR markers

Primers were designed successfully for 3,059 SSR loci using Primer Premier 3.0. However, the remaining 2,167 SSR loci did not have enough flanking sequences for primer design. SSR markers developed in this study were designated with the prefix ‘NNFB_’ and a number (NNFB_1 – NNFB_3059). Primer sequences are presented in **Table S2**.

We randomly selected 575 primer pairs for synthesis and validation. DNA fragments were successfully amplified from 514 primer pairs (89.4%), but failed from the rest of primer pairs at various annealing temperatures and Mg^2+^ concentrations (**Table S3**). PCR amplification resulted in 217 SSRs (42.2%) that were polymorphic for seven representative accessions of *N. nucifera* and one accession of *N. lutea*. In fact, of the 217 polymorphic primers, 109 primer pairs were polymorphic among the *Nelumbo* accessions, and 108 primer pairs were polymorphic only between *N. nucifera* and *N. lutea*, suggesting that the 108 markers had no allelic polymorphism among the *N. nucifera* accessions (**Table S3**). EST sequences, from which all 217 polymorphic markers were designed and developed, are listed in **Dataset S1**.

The 109 SSR polymorphic markers among the *Nelumbo* accessions in the study were used to genotype a sample of 44 accessions plants representing diverse genotypes of *Nelumbo* (**Table S4**). A total of 394 alleles were identified. The number of alleles per locus varied from 2 to 9, with an average of 3.7 alleles per locus. Polymorphic information content (PIC) ranged from 0.6 for NNFB_1635 to 0.9 for NNFB_1280 with an average value of 0.8 per marker (**Table S5**) suggesting that the EST-SSRs uncovered in this study were highly polymorphic.

### Diversity analysis and genetic relationship revealed by EST-SSRs

**Figure 6:**
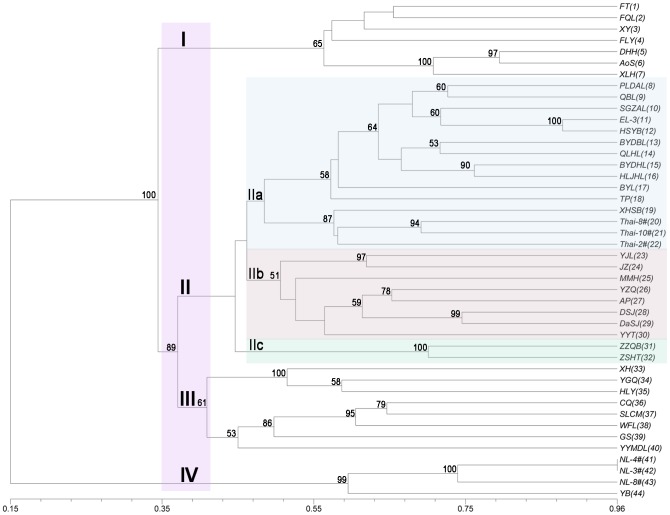
Genetic diversity analysis among *Nelumbo* accessions based on genic SSR markers. The dendrogram shows the genetic relationships among 44 accessions of *Nelumbo*. Scale at the bottom of the dendrogram indicates the level of similarity between the genotypes and the numbers on the nodes are bootstrap values (>50%) from 1000 replicates.

Jaccard’s similarity coefficients were calculated for pairwise combinations of all genotypes and a dendrogram was constructed to resolve the members of four distinct groups, I, II, III and IV, at a cut-off similarity coefficient of 0.39 (**Figure 6**). All genotypes of *N. nucifera* clustered in Group I and Group II (**Figure 6**, **Table S4**). GroupI contained seven *N. nucifera* accessions. Group II contained twenty-five accessions of *N. nucifera* and was subdivided into three distinct clusters (IIa, IIb and IIc) at a cut-off similarity coefficient of 0.47, which strongly reflected the derivation of the *N. nucifera* accessions as wild or cultivars. Fifteen samples of wild, rhizome, thousand-petalled and tropical lotus types were clustered into Subgroup IIa, all of which were genotypes of wild accessions with different geographic locations in either China or Thailand, except for two flower-lotus cultivars (BYL and TP), one rhizome-lotus cultivar (EL-3) and one tropical cultivar (XHSB). Subgroup IIb contained eight flower lotus cultivars, and two red flower lotus cultivars with a number of common morphological traits clustered in Subgroup IIc. All genotypes of *N. lutea* and their interspecific hybrids with *N. nucifera* were clustered in Group III and Group IV **(** **Figure 6**, **Table S**4). Group III contained eight Asian-American hybrids and Group IV was composed of four wild *N. lutea* accessions.

## Discussion

The transcriptome of the flower buds from three accessions of *N. nucifera* was deep sequenced and analyzed. This is the first paper reporting large-scale transcript data from flower-buds of *Nelumbo*. This transcriptome information provides a significant addition to the existing genomic or functional-genomic resources of lotus. Genic SSR markers developed in this study will enrich the number of SSR markers and facilitate basic and applied genomic research in lotus.

### Transcriptome sequencing and assembly

Transcriptome sequencing is an important approach for gene discovery, expression pattern identification, and molecular marker development [28]. The next generation sequencing (NGS) technologies including Roche/454, Solexa/Illumina and ABI /SOLiD platforms have made it possible to generate large-scale genome resources at a relatively low cost [39]–[41]. Among these NGS methods, 454 GS-FLX Titanium provides a rapid, efficient and cost-effective method for genomic resource enrichment by generating ESTs with larger individual read lengths up to 500 bp [42]. This method has been widely utilized for *de novo* transcriptome sequencing and assembly in many organisms [24], [26], [31], [35], [42]–[47]. In this study, we used the 454 GS-FLX technology platform to generate a total of 1.34 million reads (about 0.45 GB) from a mixed flower-bud cDNA pool. This tissue-specific transcriptome study will provide good reference data for expression profiling of tissue-specific genes, especially in non-model plants [47]. Therefore, these large-scale ESTs generated in our study will provide more comprehensive flower-bud transcriptome information and facilitate the identification of genes involved in lotus growth and development, especially in flower development.

Some previous studies indicated that the 454 GS-FLX Titanium technology provided larger read lengths, but fewer relatively numbers of reads than the Illumina technology [31]. This has been verified in our study. The number of reads (about 0.45 GB) in our study was less than that obtained by Illumina sequencing of other lotus tissues (about 1.2–2.9 GB), previously deposited in NCBI public databases. Long read lengths permit assembly of larger contigs [42]. A total of 715,559 (53.3%) reads were more than 400 bp in our study, and the average length of contigs assembled was 620 bp, which is considerably longer than that derived from previous studies, such as 276 [29], 440 [48], 521 [49], 550 [39], and 605 bp [50].

For sequence annotation, 50.1% (34,341) of 68,593 unigenes in our dataset showed at least one significant homolog to genes in other species by BLASTx targeting NCBI Non-Redundant protein database. The higher percentage of hits was partially due to the increased number of long sequences in our unigene database (506 bp on average). The remaining unigenes (about 50%) could not be functionally annotated because they were matched to a protein of unknown/uncharacterized function or had no BLAST matches in the database. The ability to detect significant sequence similarities depends on the length of the query sequence in most cases. Some previous studies showed that longer unigenes were more likely to have BLAST matches in protein databases [33], [51]. Our study demonstrated that 83.3% of the unigenes over 1000 bp in length matched a homolog, whereas only 19.3% of the unigenes shorter than 300 bp matched homologs. In addition, only limited genomic and transcriptomic information are available for lotus, hence many lotus genes are not included in current public databases.

### EST-SSR frequency and distribution in the lotus transcriptome

Polymorphic SSR markers play important roles in genetic diversity, population genetics, gene cloning, map construction, comparative genomics, and MAS breeding, etc. Although about five hundred SSR markers have been developed for lotus, only 39 markers are genic SSRs [7], [11]. This limited number of SSR markers blocked both basic and applied genomics research in lotus. Deep transcriptome sequencing provides a good resource for the development of numerous SSRs because of the quantity of sequences it generates. Markers based on transcriptome sequences are more useful for detection of functional variation and gene-based analysis [29]. In this study, a total of 6,086 potential SSR markers were identified from 5,482 unigene sequences (**Table 2**), and 8.0% of the transciptome sequences possessed SSR loci. This rate falls into the range of frequencies reported for other dicotyledonous species (2%–17%) [52]. The SSR frequency is different among various species, in part because of arithmetical methods for SSR detection [28], search parameters for exploring SSRs [29], and genome size or structure [53], [54]. SSR frequency in lotus is higher than barley (2.8%), *Epimedium* (3.7%), wheat (7.4%), and pigeonpea (7.6%), but lower than sesame (8.9%) and *Amorphophallus* (11.8%) [28]–[29], [35], [55]–[57]. The abundance of SSRs in lotus is one SSR locus per 5.7 kb (**Table 2**), compared to 3.4 kb in rice, 3.5 kb in radish, 3.6 kb in *Amorphophallus*, 5.4 kb in wheat, 7.4 kb in soybean, and 8.4 kb in pigeon pea [29], [33], [35]. The difference in SSR abundance could partially account for the size of unigene assembly dataset, different search criteria, and data mining tools [21], [34].

Di-nucleotide repeats were the most frequent SSR motif type (**Figure 5a**), representing 65.2% of SSR markers identified in this study. This is consistent with the previous reports in *Arabidopsis*, peanut, canola, sugar beet, cabbage, soybean, pigeon pea, sunflower, rubber tree, sesame, sweet potato, pea, grape, and *Amorphophallus* [28]–[29], [35], [52], [58]. Mononucleotide repeat motifs were excluded in our analysis because of the potential sequencing errors. Among the di-nucleotide repeats, AG/CT (57.9%), also found in other plant species [28], [58]–[59], was the most frequent motif in our transcriptome dataset. Previous studies suggested that the tri-nucleotide AAG/CTT is a common motif and CCG/CGG is rare in dicotyledonous plants [52], [59]. This phenomenon was confirmed by our studies showing that the most common tri-nucleotide motif was GAA/AGA/AAG (13.3%) and that C/G-rich (0.5%) motifs were rare. Moreover, the most frequent motif and their types of genic SSRs in our study are in agreement with that observed in genomic-SSRs from Yang *et al.* in lotus [7]. The complete list of SSR (3,059) markers and their corresponding primer pair information were provided in **Table S2.**

### Polymorphism of EST-SSR markers and evaluation of genetic relationships

Genetic diversity analyses of lotus germplasm has mostly depended on RAPD, ISSR, AFLP, and genomic SSR markers [10]–[15]. Only 39 EST-SSR markers for lotus have been developed previously [11]. By deep transcriptome sequencing, we identified a more extensive genic SSR marker set for lotus.

Genic SSRs are useful and often preferred for locating coding regions of the genome, and frequently show a high degree of transferability to the related species [29], [60]. To validate our SSR markers, a total of 575 primer pairs were synthesized and tested, of which 514 primers (89.4%) successfully yielded amplicons in three accessions of *Nelumbo* (**Table S4**). This result was similar to the success rate of 60%–90% amplification previously reported [27], [29], [59]. Lack of amplicon production by other primer pairs may have been due to the location of the primers across splice-sites, large introns, or poor-quality sequences [34]. Genic SSRs are generally less polymorphic than genomic SSRs because of greater sequence conservation in the transcribed regions [59], but the use of genic SSRs developed in our study showed a high level of polymorphism. Previous studies on the genetic diversity of *Nelumbo* using genomic-SSRs reported an average of 3.3–5.8 alleles per locus with average PIC values of 0.3 – 0.5 [13]–[15], [35]–[37]. One study of genic SSR markers in lotus reported the mean number of alleles per marker as 2.7 and an average PIC value of 0.3 [11]. In this study, we observed a similar average of 3.7 alleles per locus and a higher average PIC value of 0.8 by using genic SSR markers. We attributed this to the higher coverage depth we achieved. Such depth generally produces larger contigs including UTRs that are more polymorphic [35] and the use of diverse genotypes of *Nelumbo* including wild lotus species, special interspecific hybrids and tropical accessions for diversity analysis.

A dendrogram showed that *N*. *lutea* accessions in Group III and their interspecific hybrids with *N. nucifera* in Group IV were clearly separated from *N. nucifera* accessions in GroupI and GroupII. Results confirmed that *N*. *lutea* is genetically distinct from *N. nucifera*, as reported previously with various types of molecular markers [7]–[15]. Wild accessions of *N. nucifera* that clustered in SubgroupIIa were distinct from *N. nucifera* cultivars in GroupI and SubgroupsIIb and IIc, suggesting that the cultivars and wild plants have experienced divergence as a result of advances in modern agriculture and changes in environment [11]. Genotypes of both Chinese and Thai lotus belong to *N. nucifera*; however, they clustered in different groups. A total of eight tropical accessions were used to evaluate their genetic variations, of which four cultivars were placed in GroupI. Previous studies have indicated that these tropical Thai accessions selected from Southeast Asia germplasm belong to a different ecotype and were genetically different from the temperate-type Chinese lotus accessions [10], [15], a finding also supported by our study. Other three wild Thai accessions and one Thai cultivar (XHSB) were clustered together with Chinese wild accessions in SubgroupsIIa. The potential reason is that genic SSR markers from the transcribed portion of the genome are more evolutionarily conserved within and across related species, and different wild accessions may share similar gene sequences [34]. The analysis of genotypic diversity based on the genic SSR markers in this study clearly illustrates the existence of several clusters within *Nelumbo* germplasm (**Figure 6**). However, several accessions, particularly some cultivars of *N. nucifera*, were clustered in different Groups or Subgroups and lacked a clear pattern related to morphological characteristics. This result could be explained by three reasons: 1) the sample number of accessions for diversity analysis is not large enough to show a clear pattern, 2) the cultivars selected by us could harbor high genetic diversity caused by cross-breeding [15], 3) some accessions could have been misclassified by previous studies using morphological characteristics as the classification standards.

## Conclusions

In this study, we generated more than 1.34 million lotus cDNA sequences from flower buds of three *N. nucifera* accessions using 454 GS-FLX Titanium technology. This is the first report on the transcriptome of lotus flower buds. The ESTs generated in this report are significant additions to existing genomic and functional genomics resources of lotus. These ESTs will facilitate annotation of the lotus genome and identification of genes involved in lotus growth and development, especially those involved in flower development. A total of 3,059 SSR loci were successfully designed the primer pairs in the study, of which 575 were validated for amplification and polymorphism. Using the validated primers, genetic diversity across 44 accessions of *Nelumbo* was examined. These identified many genic SSR markers that will be valuable resources for genetic diversity analysis, construction of linkage map, genes mapping, and MAS breeding in lotus.

## Materials and Methods

### Plant materials and DNA extraction

Young flower-buds (35 - 40 mm in length) of three accessions of *N. nucifera* (**Table S6**) were collected for RNA extraction and transcriptome sequencing. Forty-four accessions, representing diverse genotypes of *Nelumbo,* were used for marker validation and genetic diversity analysis. Most of the plant materials used in this study were produced by clonal propagation in pools at Shanghai Chenshan Botanical Garden (Shanghai, China), to prevent genetic contamination of different cultivars and species. Detailed information on plant materials is listed in **Table S4**.

Genomic DNA was extracted using the DNAsecure Plant kit (TIANGEN Inc. Beijing, China) following the manufacturer’s protocol. DNA samples were dissolved in TE buffer (pH 8.0) and visualized on 0.8% agarose gels in 1×TAE. DNA purity and concentration was measured with a NanoDrop 2000c UV-Vis spectrophotometer (Thermo Fisher Scientific Inc., USA). DNA was adjusted to a final concentration of 30 ng·μl^−1^ and stored at −20°C until use.

### cDNA preparation and 454 sequencing

Field-collected young flower buds of three *N. nucifera* accessions were picked and immediately frozen in liquid nitrogen and stored at −80°C. Total RNA was extracted using the TRIzol Reagent (Invitrogen). Equal quantities of RNA from the flower buds of three accessions were blended to create a mixed pool for maximizing the diversity of transcriptional units. cDNA synthesis was performed using the Clontech SMART system (Clontech Lab, inc. CA, USA). For 454 sequencing, the cDNA library was prepared according to the manufacturer’s protocol using the Roche GS-FLX Titanium General Library Preparation Kit. The quality of cDNA was evaluated using the Agilent Bioanalyzer 2100 (Agilent Technology, inc. USA). The pooled library was sequenced in a full 454 plate run on the GS-FLX Titanium platform following standard procedures. The transcriptome dataset was deposited in the Gene Expression Omnibus database with an accession number of GSE57601.

### Assembly and functional annotation

Raw data from 454 sequencing were pre-processed to remove adaptor-ligated regions, primers and very short sequences (<50 bp) by Seqclean (v86_64) [61] and to trim low-quality regions by the LUCY program (v2.19) [62]. Cleaned and qualified reads were then assembled *de novo* in Newbler (v2.5.3) with optimal parameters [31], [63], [64]. The assembled unique sequences were separately combined and clustered with CD-HIT 4.0 [65], [66]. Sequences of similarity with > 95% identity were clustered into one class and the longest sequence of each clustered class was treated as a unigene.

Putative unigenes were compared against the NCBI Non-Redundant protein database (http://www.ncbi.nlm.nih.gov/) using BLASTx with an E-value cut-off of 1e^−5^. The procedure was used to provide a specific functional annotation for each unigene, based on sequence similarity. The best alignment results were selected to annotate the unigenes. Functional classifications of the annotated unigenes were based on GO terms using Blast2GO program [67] and KEGG pathway using custom Perl script.

### Detection of SSR markers and primer design

All unigenes obtained in the study were used to detect SSR loci with MIcroSAtellite Perl script (MISA, http://pgrc.ipk-gatersleben.de/misa). SSR loci were considered to contain two to six nucleotide motifs with minimum repeats of 6, 5, 5, 5 and 5, respectively. Primer 3.0 program [68] was used for designing PCR primer pairs based on the following parameters: (1) primer length ranging from 18 bp to 27 bp with an optimum size of 20 bp, (2) melting temperatures (Tm) between 57°C and 63°C with 60°C as optimum, (3) GC content between 40% and 60%, and (4) PCR product size ranging from 100 bp to 280 bp.

### PCR amplification and evaluation of SSR polymorphism

A total of 575 primers were selected from newly designed SSR markers to evaluate SSR polymorphisms. All of 575 SSRs were first tested for PCR amplification using genomic DNA of three accessions of *Nelumbo* to amplify the target band and optimize the annealing temperature. The optimized SSRs were then used to detect polymorphisms in eight lotus accessions (seven representative accessions of *N. nucifera* and one of *N. lutea*). Polymorphic SSRs were evaluated for genetic diversity analysis in forty-four accessions of *Nelumbo*. PCR amplification for SSRs was carried out in a 10 µl reaction volume with the following conditions: 94°C for 5 min, followed by 30 cycles at 94°C for 30 s, 52°C for 30 s, and 72°C for 30 s and a final extension at 72°C for 5 min. The amplification products were separated on 6% denatured polyacrylamide gels with 1× TBE buffer at a constant power of 50 W for 1.5 h. After electrophoresis, the gel was silver-stained [69] and photographed with a digital camera (Nikon D90). All primers were synthesized by Sangon Biological Engineering Technology & Service Co. (Shanghai, China).

### Data scoring and genetic analysis

Differently sized fragments of EST-SSR were scored as unique alleles and recorded manually in binary format (allele presence  =  1, allele absence  =  0). The binary matrix file was utilized to calculate pairwise Jaccard’s similarity coefficients. Based on the similarity matrix, all 44 accessions were clustered using UPGMA analysis and the SHAN clustering program by NTSYS-pc v2.11 [70]. The value of the polymorphic information content (PIC) for each EST-SSR primer was calculated for all 44 *Nelumbo* cultivars, as previously described [71]. Bootstrapping analysis was carried out using FREETREE software. Bootstrap values (> 50%) estimated by 10, 000 replicates are considered significant and are indicated on the dendrogram.

## Supporting Information

Table S1 **Transcripts related to flower development in** ***Nelumbo*** **.**
(XLSX)

Table S2 **List of EST-SSR markers identified in the study.** All information about the primer names, unigene ID, repeat motifs, primer sequences, expected product size (bp) and annealing temperature, and putative gene function based on BLASTx similarity search are listed.
(XLSX)

Table S3 **Details of 575 selected EST-SSR markers for polymorphism validation.**
(XLSX)

Table S4 ***Nelumbo*** **germplasm for the validation and genetic diversity analysis with EST-SSRs.** Detailed information of each individual including the accession names, species, type, place of collection and group is listed.
(XLS)

Table S5 **Characteristics of 109 polymorphic markers used for genetic diversity analysis.**
(XLSX)

Table S6 **Information on the three accessions of** ***N. nucifera*** **employed for deep transcriptome sequencing.**
(XLS)

Dataset S1 **EST sequences of 152 genes and 217 polymorphic markers identified in the study.**
(TXT)

## References

[1] Angiosperm phylogeny group (2009) An update of the Angiosperm Phylogeny Group classification for the orders and families of flowering plants: APG III. Bot J Linn Soc 161: 105–121.

[2] Diao Y, Chen L, Yang G, Zhou M, Song Y, et al (2006) Nuclear DNA C-values in 12 species in Nymphaeales. Caryologia 59(1): 25–30.

[3] Ming R, VanBuren R, Liu Y, Yang M, Han Y, et al (2013) Genome of the long-living sacred lotus (*Nelumbo nucifera* Gaertn.). Genome Biol 14: R41. 23663246 10.1186/gb-2013-14-5-r41 PMC4053705

[4] Huang X, Chen J, Huang G (1992) Preliminary studies on biosystematic relationship between the two *Nelumbo* species. Acta Hortic Sin 19(2): 164–170.

[5] Shen-Miller J (2002) Sacred lotus, the long-living fruits of China Antique. Seed Sci Res 12: 131–143.

[6] Wang QC, Zhang XY (2005) Colored Illustration of Lotus Cultivars in China. Beijing: China Forestry Publishing House.

[7] Yang M, Han Y, Robert V, Ming R, Xu L, et al (2012) Genetic linkage maps for Asian and American lotus constructed using novel SSR markers derived from the genome of sequenced cultivar. BMC Genomics 13: 653. 23170872 10.1186/1471-2164-13-653 PMC3564711

[8] Chen Y, Zhou R, Lin X, Wu K, Qian X, et al (2008) ISSR analysis of genetic diversity in sacred lotus cultivars. Aquat Bot 89: 311–316.

[9] Kubo N, Hirai M, Kaneko A, Tanaka D, Kasumi K (2009) Classification and diversity of sacred and American *Nelumbo* species: the genetic relationships of flowering lotus cultivars in Japan using SSR markers. Plant Genetic Resources 7(03): 260–270.

[10] Li Z, Liu X, Robert W, Niran J, Zhou M, et al (2010) Genetic diversity and classification of *Nelumbo* germplasm of different origins by RAPD and ISSR analysis. Sci Hortic 125: 724–732.

[11] Pan L, Xia Q, Quan Z, Liu H, Ke W, et al (2010) Development of novel EST-SSRs from sacred lotus (*Nelumbo nucifera* Gaertn.) and their utilization for the genetic diversity analysis of *N. nucifera* . J Hered 101(1): 71–82. 19666746 10.1093/jhered/esp070

[12] Fu J, Xiang Q, Zeng X, Yang M, Wang Y, et al (2011) Assessment of the genetic diversity and population structure of lotus cultivars grown in China by amplified fragment length polymorphism. J Am Soc Hortic Sci 136(5): 1–11.

[13] Pan L, Quan Z, Hu J, Wang G, Liu S, et al (2011) Genetic diversity and differentiation of lotus (*Nelumbo nucifera*) accessions assessed by simple sequence repeats. Ann Appl Biol 159(3): 428–441.

[14] Hu J, Pan L, Liu H, Wang S, Wu Z, et al (2012) Comparative analysis of genetic diversity in sacred lotus (*Nelumbo nucifera* Gaertn.) using AFLP and SSR markers. Mol Biol Rep 39(4): 3637–3647. 21735103 10.1007/s11033-011-1138-y

[15] Liu Y, Yang M, Xiang Q, Xu L, Zeng X, et al (2012) Characterization of microsatellite markers and their application for the assessment of genetic diversity among lotus accessions. J Am Soc Hortic Sci 137: 180–188.

[16] Zhang XY, Chen LQ, Wang QC (2011) New lotus flower cultivars in China. Beijing: China forestry Publishing House.

[17] VanBuren R, Walters B, Ming R, Min X (2013) Analysis of expressed sequence tags and alternative splicing genes in sacred lotus (*Nelumbo nucifera* Gaertn.). POJ 6(4): 311–317.

[18] Yang M, Zhu L, Xu L, Pan C, Liu Y (2014) Comparative transcriptomic analysis of the regulation of flowering in temperate and tropical lotus (*Nelumbo nucifera*) by RNA-Seq. Ann Appl Biol 165: 73–95.

[19] Guo S, Zheng Y, Joung J, Liu S, Zhang Z, et al (2010) Transcriptome sequencing and comparative analysis of cucumber flowers with different sex types. BMC Genomics 11: 384. 20565788 10.1186/1471-2164-11-384 PMC2897810

[20] Garg R, Patel K, Tyagi AK, Jain M (2011) *De novo* assembly of chickpea transcriptome using short reads for gene discovery and marker identification. DNA Res 18: 53–63. 21217129 10.1093/dnares/dsq028 PMC3041503

[21] Raju NL, Gnanesh BN, Lekha P, Jayashree B, Pande S, et al (2010) The first set of EST resource for gene discovery and marker development in pigeonpea (*Cajanus cajan* L.). BMC Plant Biol 10: 45. 20222972 10.1186/1471-2229-10-45 PMC2923520

[22] Yang L, Ding G, Lin H, Cheng H, Kong Y, et al (2013) Transcriptome analysis of medicinal plant *Salvia miltiorrhiza* and identification of genes related to tanshinone biosynthesis. PLoS ONE 8(11): e80464. 24260395 10.1371/journal.pone.0080464 PMC3834075

[23] Libault M, Farmer A, Joshi T, Takahashi K, Langley RJ, et al (2010) An integrated transcriptome atlas of the crop model *Glycine max*, and its use in comparative analyses in plants. Plant J 63: 86–99. 20408999 10.1111/j.1365-313X.2010.04222.x

[24] Parra-González LB, Aravena-Abarzúa GA, Navarro-Navarro CS, Udall J, Maughan J, et al (2012) Yellow lupin (*Lupinus luteus* L.) transcriptome sequencing: molecular marker development and comparative studies. BMC Genomics 13: 425. 22920992 10.1186/1471-2164-13-425 PMC3472298

[25] Eveland AL, McCarty DR, Koch KE (2008) Transcript profiling by 3'-untranslated region sequencing resolves expression of gene families. Plant Physiol 146: 32–44. 18024554 10.1104/pp.107.108597 PMC2230554

[26] Blanca J, Canizares J, Roig C, Ziarsolo P, Nuez F, et al (2011) Transcriptome characterization and high throughput SSRs and SNPs discovery in *Cucurbita pepo* (Cucurbitaceae). BMC Genomics 12: 104. 21310031 10.1186/1471-2164-12-104 PMC3049757

[27] Wang Z, Fang B, Chen J, Zhang X, Luo Z, et al (2010) *De novo* assembly and characterization of root transcriptome using Illumina paired-end sequencing and development of cSSR markers in sweetpotato (*Ipomoea batatas*). BMC Genomics 11: 726. 21182800 10.1186/1471-2164-11-726 PMC3016421

[28] Wei W, Qi X, Wang L, Zhang Y, Hua W, et al (2011) Characterization of the sesame (*Sesamum indicum* L.) global transcriptome using Illumina paired-end sequencing and development of EST-SSR markers. BMC Genomics 12: 451. 21929789 10.1186/1471-2164-12-451 PMC3184296

[29] Zheng X, Pan C, Diao Y, You Y, Yang C, et al (2013) Development of microsatellite markers by transcriptome sequencing in two species of *Amorphophallus* (Araceae). BMC Genomics 14: 490. 23870214 10.1186/1471-2164-14-490 PMC3737116

[30] Nishiyama T, Fujita T, Shin-I T, Seki M, Nishide H, et al (2003) Comparative genomics of *Physcomitrella patens* gametophytic transcriptome and *Arabidopsis thaliana*: Implication for land plant evolution. P Natl Acad Sci 100: 8007–8012. 10.1073/pnas.0932694100 PMC164703 12808149

[31] Niu S, Li Z, Yuan H, Chen X, Li Y, et al (2013) Transcriptome characterisation of *Pinus tabuliformis* and evolution of genes in the *Pinus* phylogeny. BMC Genomics 14: 263. 23597112 10.1186/1471-2164-14-263 PMC3640921

[32] Powell W, Machray GC, Provan J (1996) Polymorphism revealed by simple sequence repeats. Trends Plant Sci 1: 215–222.

[33] Wang S, Wang X, He Q, Liu X, Xu W, et al (2012) Transcriptome analysis of the roots at early and late seedling stages using Illumina paired-end sequencing and development of EST-SSR markers in radish. Plant Cell Rep 31: 1437–1447. 22476438 10.1007/s00299-012-1259-3

[34] Varshney RK, Graner A, Sorrells ME (2005) Genic microsatellite markers in plants: features and applications. Trends Biotechnol 23(1): 48–55. 15629858 10.1016/j.tibtech.2004.11.005

[35] Dutta S, Kumawat G, Singh BP, Gupta DK, Singh S, et al (2011) Development of genic-SSR markers by deep transcriptome sequencing in pigeonpea [*Cajanus cajan* (L.) Millspaugh]. BMC Plant Biol 11: 17. 21251263 10.1186/1471-2229-11-17 PMC3036606

[36] Kubo N, Hirai M, Kaneko A, Tanaka D, Kasumi K (2009) Development and characterization of simple sequence repeat (SSR) markers in the water lotus (*Nelumbo nucifera*). Aquat Bot 90(2): 191–194.

[37] Pan L, Quan Z, Li S, Liu H, Huang X, et al (2007) Isolation and characterization of microsatellite markers in the sacred lotus (*Nelumbo nucifera* Gaertn.). Mol Ecol Notes 7(6): 1054–1056.

[38] Tian H, Chen X, Wang J, Xue J, Wen J, et al (2008) Development and characterization of microsatellite loci for lotus (*Nelumbo nucifera*). Conserv Genet 9(5): 1385–1388.

[39] Hiremath PJ, Farmer A, Cannon SB, Woodward J, Kudapa H, et al (2011) Large-scale transcriptome analysis in chickpea (*Cicer arietinum* L.), an orphan legume crop of the semi-arid tropics of Asia and Africa. Plant Biotechnol J 9: 922–931. 21615673 10.1111/j.1467-7652.2011.00625.x PMC3437486

[40] Mardis ER (2008) The impact of next-generation sequencing technology on genetics. Trends Genet 24: 133–141. 18262675 10.1016/j.tig.2007.12.007 PMC2680276

[41] Varshney RK, Nayak SN, May GD, Jackson SA (2009) Next generation sequencing technologies and their implications for crop genetics and breeding. Trends Biotechnol 27: 522–530. 19679362 10.1016/j.tibtech.2009.05.006

[42] Kaur S, Cogan NO, Pembleton LW, Shinozuka M, Savin KW, et al (2011) Transcriptome sequencing of lentil based on second-generation technology permits large-scale unigene assembly and SSR marker discovery. BMC Genomics 12: 265. 21609489 10.1186/1471-2164-12-265 PMC3113791

[43] Meyer E, Aglyamova GV, Wang S, Buchanan-Carter J, Abrego D, et al (2009) Sequencing and de novo analysis of a coral larval transcriptome using 454 GSFlx. BMC Genomics 10: 219. 19435504 10.1186/1471-2164-10-219 PMC2689275

[44] Zagrobelny M, Scheibye-Alsing K, Jensen NB, Moller BL, Gorodkin J, et al (2009) 454 pyrosequencing based transcriptome analysis of *Zygaena filipendulae* with focus on genes involved in biosynthesis of cyanogenic glucosides. BMC Genomics 10: 574. 19954531 10.1186/1471-2164-10-574 PMC2791780

[45] Hou R, Bao ZM, Wang S, Su HL, Li Y, et al (2011) Transcriptome sequencing and *de novo* analysis for Yesso scallop (*Patinopecten yessoensis*) using 454 GS FLX. PLoS ONE 6: e21560. 21720557 10.1371/journal.pone.0021560 PMC3123371

[46] Liao X, Cheng L, Xu P, Lu G, Wachholtz M, et al (2013) Transcriptome analysis of Crucian carp (*Carassius auratus*), an important aquaculture and Hypoxia-tolerant species. PLoS ONE 8(4): e62308. 23630630 10.1371/journal.pone.0062308 PMC3632525

[47] Zhou Y, Gao F, Liu R, Feng J, Li H (2012) *De novo* sequencing and analysis of root transcriptome using 454 pyrosequencing to discover putative genes associated with drought tolerance in *Ammopiptanthus mongolicus* . BMC Genomics 13: 266. 22721448 10.1186/1471-2164-13-266 PMC3407029

[48] Sun C, Li Y, Wu Q, Luo H, Sun Y, et al (2010) *De novo* sequencing and analysis of the American ginseng root transcriptome using a GS FLX Titanium platform to discover putative genes involved in ginsenoside biosynthesis. BMC Genomics 11: 262. 20416102 10.1186/1471-2164-11-262 PMC2873478

[49] Lu F, Cho M, Park Y (2012) Transcriptome profiling and molecular marker discovery in red pepper, *Capsicum annuum* L. TF68. Mol Biol Rep 39: 3327–3335. 21706160 10.1007/s11033-011-1102-x

[50] Tanase1K, Nishitani C, Hirakawa H, Isobe S, Tabata S, et al (2012) Transcriptome analysis of carnation (*Dianthus caryophyllus* L.) based on next-generation sequencing technology. BMC Genomics 13: 292. 22747974 10.1186/1471-2164-13-292 PMC3411436

[51] Wang X, Luan J, Li J, Bao Y, Zhang C, et al (2010) *De novo* characterization of a whitefly transcriptome and analysis of its gene expression during development. BMC Genomics 11: 400. 20573269 10.1186/1471-2164-11-400 PMC2898760

[52] Kumpatla S, Mukhopadhyay S (2005) Mining and survey of simple sequence repeats in expressed sequence tags of dicotyledonous species. Genome 48(6): 985–998. 16391668 10.1139/g05-060

[53] Toth G, Gaspari Z, Jurka J (2000) Microsatellites in different eukaryotic genomes: survey and analysis. Genome Res 10(7): 967–981. 10899146 10.1101/gr.10.7.967 PMC310925

[54] Varshney R, Thiel T, Stein N, Langridge P, Graner A (2002) In silico analysis on frequency and distribution of microsatellites in ESTs of some cereal species. Cell Mol Biol Lett 7(2A): 537–546. 12378259

[55] Varshney R, Grosse I, Hähnel U, Siefken R, Prasad M, et al (2006) Genetic mapping and BAC assignment of EST-derived SSR markers shows non-uniform distribution of genes in the barley genome. Theor Appl Genet 113(2): 239–250. 16791690 10.1007/s00122-006-0289-z

[56] Peng J, Lapitan NLV (2005) Characterization of EST-derived microsatellites in the wheat genome and development of eSSR markers. Funct Integr Genomic 5(2): 80–96. 10.1007/s10142-004-0128-8 15650880

[57] Zeng S, Xiao G, Guo J, Fei Z, Xu Y, et al (2010) Development of a EST dataset and characterization of EST-SSRs in a traditional Chinese medicinal plant, *Epimedium sagittatum* (Sieb. Et Zucc.) Maxim. BMC Genomics 11(1): 94. 20141623 10.1186/1471-2164-11-94 PMC2829513

[58] Li D, Deng Z, Qin B, Liu X, Men Z (2012) *De novo* assembly and characterization of bark transcriptome using Illumina sequencing and development of EST-SSR markers in rubber tree (*Hevea brasiliensis* Muell. Arg.). BMC Genomics 13: 192. 22607098 10.1186/1471-2164-13-192 PMC3431226

[59] Liang X, Chen X, Hong Y, Liu H, Liu H, et al (2009) Utility of EST-derived SSR in cultivated peanut (*Arachis hypogaea* L.) and *Arachis* wild species. BMC Plant Biol 9(1): 35. 19309524 10.1186/1471-2229-9-35 PMC2678122

[60] Vendramin E, Dettori M, Giovinazzi J, Micali S, Quarta R, et al (2007) A set of EST-SSRs isolated from peach fruit transcriptome and their transportability across *Prunus* species. Mol Ecol Notes 7(2): 307–310.

[61] SeqClean program. Available: http://sourceforge.net/projects/seqclean/.

[62] Li S, Chou H (2004) LUCY2: an interactive DNA sequence quality trimming and vector removal tool. Bioinformatics 20(16): 2865–2866. 15130926 10.1093/bioinformatics/bth302

[63] Kumar S, Blaxter ML (2010) Comparing *de novo* assemblers for 454 transcriptome data. BMC Genomics 11: 571. 20950480 10.1186/1471-2164-11-571 PMC3091720

[64] Mundry M, Bornberg-Bauer E, Sammeth M, Feulner PG (2012) Evaluating characteristics of *de novo* assembly software on 454 transcriptome data: a simulation approach. PLoS ONE 7(2): e31410. 22384018 10.1371/journal.pone.0031410 PMC3288049

[65] Huang Y, Niu B, Gao Y, Fu L, Li W (2010) CD-HIT Suite: a web server for clustering and comparing biological sequences. Bioinformatics 26(5): 680–682. 20053844 10.1093/bioinformatics/btq003 PMC2828112

[66] Li W, Godzik A (2006) Cd-hit: a fast program for clustering and comparing large sets of protein or nucleotide sequences. Bioinformatics 22(13): 1658–1659. 16731699 10.1093/bioinformatics/btl158

[67] Conesa A, Gotz S, Garcia-Gomez JM, Terol J, Talon M, et al (2005) Blast2GO: a universal tool for annotation, visualization and analysis in functional genomics research. Bioinformatics 21: 3674–3676. 16081474 10.1093/bioinformatics/bti610

[68] Rozen S, Skaletsky H (2000) Primer3 on the WWW for general users and for biologist programmers. Methods Mol Biol 132(3): 365–386. 10547847 10.1385/1-59259-192-2:365

[69] Bassam B, Caetana-Anolles G, Gresshoff PM (1991) Fast and sensitive silver staining of DNA in polyacrylamide gels. Anal Biochem 196: 80–83. 1716076 10.1016/0003-2697(91)90120-i

[70] Rolf J (2000) Numerical Taxonomy and Multivariate Analysis System, version 2.11T Exeter Software. Setauket, NY, USA.

[71] Botstein D, White RL, Skolnick M, Davis RW (1980) Construction of a genetic linkage map in man using restriction fragment length polymorphisms. Am J Hum Genet 32(3): 314. 6247908 PMC1686077

